# Gene clusters for biosynthesis of mycosporine‐like amino acids in dinoflagellate nuclear genomes: Possible recent horizontal gene transfer between species of Symbiodiniaceae (Dinophyceae)

**DOI:** 10.1111/jpy.13219

**Published:** 2021-11-26

**Authors:** Eiichi Shoguchi

**Affiliations:** ^1^ Marine Genomics Unit Okinawa Institute of Science and Technology Graduate University Onna Okinawa 904‐0495 Japan

**Keywords:** Symbiodiniaceae genomes, gene cluster, horizontal gene transfer, Symbidoniaceae–Symbiodiniaceae interactions, diversified MAAs, coral bleaching, *Symbiodinium*, *Durusdinium*, gene expression regulation

## Abstract

Global warming increases the temperature of the ocean surface, which can disrupt dinoflagellate‐coral symbioses and result in coral bleaching. Photosynthetic dinoflagellates of the family Symbiodiniaceae include bleaching‐tolerant and bleaching‐sensitive coral symbionts. Therefore, understanding the molecular mechanisms for changing symbiont diversity is potentially useful to assist recovery of coral holobionts (corals and their associated microbes, including multiple species of Symbiodiniaceae), although sexual reproduction has not been observed in the Symbiodiniaceae. Recent molecular phylogenetic analyses estimate that the Symbiodiniaceae appeared 160 million years ago and diversified into 15 groups, five genera of which now have available draft genomes (i.e., *Symbiodinium*, *Durusdinium*, *Breviolum*, *Fugacium*, and *Cladocopium*). Comparative genomic analyses have suggested that crown groups have fewer gene families than early‐diverging groups, although many genes that were probably acquired via gene duplications and horizontal gene transfers (HGTs) have been found in each decoded genome. Because UV stress is likely a contributor to coral bleaching, and because the highly conserved gene cluster for mycosporine‐like amino acid (MAA) biosynthesis has been found in thermal‐tolerant symbiont genomes, I reviewed genomic features of the Symbiodiniaceae, focusing on possible acquisition of a biosynthetic gene cluster for MAAs, which absorb UV radiation. On the basis of highly conserved noncoding sequences, I hypothesized that HGTs have occurred among members of the Symbiodiniaceae and have contributed to the diversification of Symbiodiniaceae–host relationships. Finally, I proposed that bleaching tolerance may be strengthened by multiple MAAs from both symbiotic dinoflagellates and corals.

**Abbreviations**
GMC oxidoreductase: glucose‐methanol‐choline oxidoreductase
HGT: horizontal gene transfer
MAAs: mycosporine‐like amino acids

Symbiotic dinoflagellates of the family Symbiodiniaceae are well‐known photosynthetic partners of corals and other nonphotosynthetic hosts in subtropical and tropical shallow waters, where they comprise essential components of coral reef ecosystems (Coffroth and Santos 2005, Brodie et al. 2017, LaJeunesse et al. 2018). Using molecular phylogenetic analyses, the family Symbiodiniaceae (previously the genus *Symbiodinium*) has been classified into 15 major groups, of which 11 were assigned to that genus (Fig. 1a; LaJeunesse et al. 2018, Nitschke et al. 2020, LaJeunesse et al. 2021, Pochon and LaJeunesse 2021). Members of the Symbiodiniaceae are hosted by ciliates, foraminiferans, sponges, cnidarians, acoels, and mollusks (Hikosaka‐Katayama et al. 2012, Pochon et al. 2014, LaJeunesse et al. 2018, Mies 2019). Although many Symbiodiniaceae–host relationships and some free‐living Symbiodiniaceae have been reported (Fig. 1a; Carlos et al. 1999, Hirose et al. 2008, Yamashita and Koike 2013, Fujise et al. 2021), recent molecular‐level studies have focused on symbiotic relationships between symbiotic dinoflagellates and corals (Davy et al. 2012, Cunning and Baker 2013, McIlroy and Coffroth 2017, Reich et al. 2017, González‐Pech et al. 2019). In this era of rapid global climate change, disruption of these relationships has resulted in coral bleaching (Smith et al. 2005) and subsequent coral mortality, leading to discussions of coral reef recovery (Loya et al. 2001, Baker et al. 2008, Dixon et al. 2015, Prada et al. 2016).

**Fig. 1:**
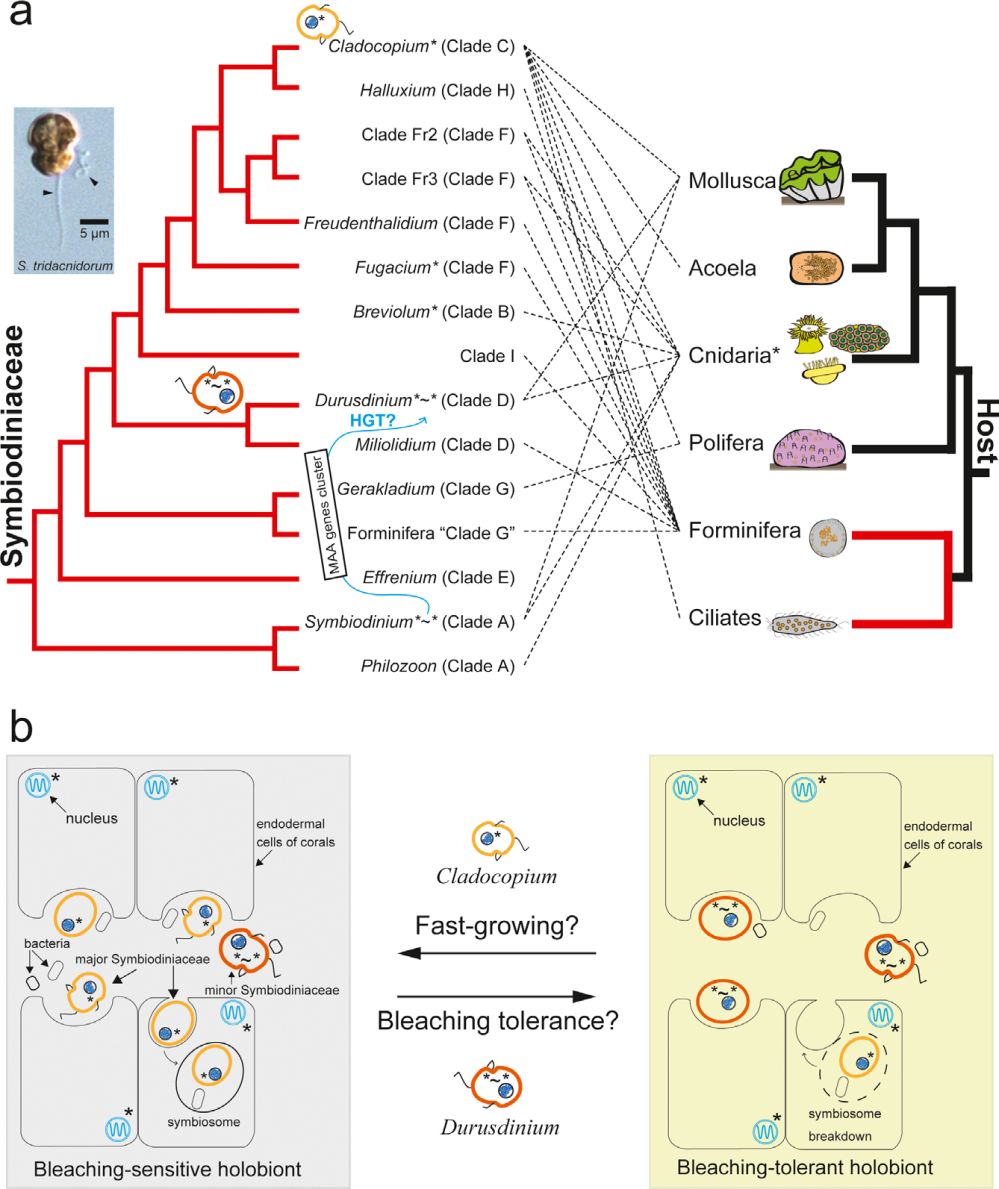
Known symbiotic connections between dinoflagellates of the family Symbiodiniaceae and hosts. (a) Left shows 15 groups of Symbiodiniaceae (Lajeunesse et al. 2018). The inset image is *Symbiodinium tridacnidorum* Y106 (NIES‐4076), showing two flagella (arrowheads), the genome of which has a gene cluster for mycosporine‐like amino acid (MAA) biosynthesis. Right indicates hosts for Symbiodiniaceae. Dashed lines show Symbiodiniaceae‐host connections that have been reported. Genera of the Symbiodiniaceae with two asterisks and a tilde (*˜*) include a sequenced genome that has a MAA biosynthetic gene cluster. The blue arrow between *Symbiodinium* and *Durusdinium* indicates the possibility that the MAA biosynthetic gene cluster may have been shared via a recent horizontal gene transfer. *Cladocopium*, *Fugacium*, and *Breviolum* with asterisks (*) have some MAA biosynthetic genes that are probably not clustered (Liu et al. 2018). Host cnidarians with asterisks (*) include corals and sea anemones that have MAA biosynthetic genes (Shinzato et al. 2011, Baumgarten et al. 2015). Red branches show the lineage of unicellular eukaryotes that may have participated in a red algal secondary endosymbiotic event (Bhattacharya et al. 2004). (b) A hypothesis for changing from bleaching‐sensitive holobionts to bleaching‐tolerant holobionts with thermally tolerant symbionts having a recently acquired MAA biosynthetic gene cluster. An illustration of a hypothesis explaining why *Durusdinium* likely contributes to bleaching resistance of corals, even though the means by which Symbiodiniaceae populations in corals respond to climate change is little known. Left shows that *Durusdinium* is a minor component in Symbiodiniaceae‐host relationships. Major Symbiodiniaceae that lack MAA biosynthetic gene clusters may contribute to fast‐growing corals (van Oppen and Medina 2020). In the right illustration, *Durusdinium*, with the MAA biosynthetic gene cluster, may enable coral holobionts to become bleaching‐tolerant (Hidaka 2016).

Collapsed symbioses between host corals and their dinoflagellate symbionts are due to rapid changes in temperature and insolation, especially UV radiation (Lesser et al. 1990, Warner et al. 1999, Loya et al. 2001, Rowan 2004). As global warming progresses, it is predicted that coral bleaching will occur more frequently (Hughes et al. 2018). Coral transplantation and other attempts at coral conservation and restoration have been performed (West and Salm 2003, Baums 2008, Shinzato et al. 2014b, Zayasu et al. 2018). Thermally tolerant coral symbionts may be important for coral conservation (Stat and Gates 2010). For example, different responses to heat stress have been observed using cultured symbiont cells (Reynolds et al. 2008, Takahashi et al. 2008, van Oppen et al. 2009, Aihara et al. 2016, Reich et al. 2021). Transplantation experiments with corals indicate that the *Durusdinium* group includes species that are more resistant to bleaching (Berkelmans and van Oppen 2006, Stat and Gates 2010). However, how the algae achieve enhanced resistance to bleaching within host corals is unknown (Ladner et al. 2012).

One strategy by which coral holobionts resist UV stress is production of water‐soluble molecules that absorb UV radiation, known as mycosporine‐like amino acids (MAAs; Shibata 1969, Shick and Dunlap 2002). However, it is unclear which of the symbiotic partners produce MAAs, because genes and enzymes for their biosynthesis were not identified until about 10 years ago. The MAA biosynthetic gene cluster was first identified in cyanobacteria by Balskus and Walsh (2010), and the decoded genome of the coral, *Acropora digitifera,* revealed that homologs of MAA biosynthetic genes are encoded in the genomes of host corals (Fig. 1; Shinzato et al. 2011). After that, draft Symbiodiniaceae genomes were reported and predicted genes were discussed (Shoguchi et al. 2013, 2018, 2021, Lin et al. 2015, Aranda et al. 2016, Liu et al. 2018, Robbins et al. 2019, Chen et al. 2020, González‐Pech et al. 2021, Yoshioka et al. 2021). We identified the MAA biosynthetic cluster in genomes of the symbiotic dinoflagellates, *Symbiodinium tridacnidorum* and *Durusdinium trenchii*. In this mini‐review, I briefly describe genomic features of these dinoflagellates and explain the gene cluster regions involved in MAA biosynthesis. For future studies, I hypothesize the possibility of horizontal gene transfers (HGTs) of these clusters between dinoflagellates and focus on a discussion of the importance of MAAs in bleaching resistance of coral holobionts.

## NUCLEAR GENOMIC FEATURES OF SYMBIOTIC DINOFLAGELLATES

Unusual nuclear features of dinoflagellates include large genome sizes and permanently condensed chromosomes (Wong 2019). Most core dinoflagellates have genomes larger than the human genome (3 Gbp; Janouškovec et al. 2017, Beedessee et al. 2020). However, some Symbiodiniaceae are thought to have smaller genomes (LaJeunesse et al. 2005, Saad et al. 2020). Transcriptomes of Symbiodiniaceae have been analyzed (Leggat et al. 2007, 2011, Bayer et al. 2012) and draft genomes of 15 strains from five genera have been published (Shoguchi et al. 2013, 2018, 2021, Lin et al. 2015, Aranda et al. 2016, Liu et al. 2018, Li et al. 2020, González‐Pech et al. 2021, Yoshioka et al. 2021). Those genomes clarified unusual gene structures and revealed many repetitive sequences (both coding and noncoding) with unknown functions, suggesting a high frequency of gene duplications. Expanded sequences include transposons, transporter genes, and repeat domain‐containing genes, such as leucine‐rich repeats and retrovirous‐related dUTPases (Lin et al. 2015, Aranda et al. 2016, Shoguchi et al. 2018). Symbiodiniaceae genes include many bacteria‐like genes (Leggat et al. 2007, Shoguchi et al. 2013), implying frequent HGTs (Fan et al. 2020). Thus far, gene family numbers in Symbiodiniaceae genomes are larger than in genomes of other major alveolates, ciliates, and apicomplexans (Shoguchi et al. 2013). Genomes in *Symbiodinium*, which is an early‐diverging sister to the other Symbiodiniaceae lineages, likely have more gene families than genomes of crown *Cladocopium*, although some *Symbiodinum* species may have fewer gene families (Shoguchi et al. 2018, González‐Pech et al. 2021). In addition to Symbiodiniaceae genomes, other recent core dinoflagellate genomes (Beedessee et al. 2020, Stephens et al. 2020) confirm that dinoflagellate genes with complex exon‐intron structures are unidirectionally arranged, suggesting polycistronic expression with spliced leader trans‐splicing (Zhang et al. 2007). Comparative analysis of Symbiodiniaceae genomes has also revealed higher GC content in *Symbiodinium* than in other Symbiodiniaceae lineages (Aranda et al. 2016, Shoguchi et al. 2018) and metabolic genes containing syntenic blocks (Liu et al. 2018). When metabolic genes acquire polycistronic expression that is likely similar to bacterial operons, higher expression levels may have been attained than is possible with monocistronic expression (Lim et al. 2011). However, such a conclusion is premature because relationships between polycistronic expression and expression regulation have yet to be determined in dinoflagellate genomes (Yang et al. 2020).

## MAA BIOSYNTHESIS IN SYMBIODINIACEAE‐CORAL SYMBIOSIS

In Symbiodiniaceae–host symbiosis, the significance of metabolic exchanges have been examined and discussed (Davy et al. 2012, Ip et al. 2020). Diversification and regulation of metabolic pathway genes in Symbiodiniaceae have likely involved specifying and maintaining symbioses with hosts (Lin et al. 2019). For example, it has been suggested that host MAAs may increase the amount of photosynthase released by the Symbiodiniaceae (Gates et al. 1995), but this has not been confirmed. On the other hand, different capacities to synthesize MAAs have been found among cultured *Symbiodinium* and other Symbiodiniaceae, including *Breviolum* and *Cladocopium* (Banaszak et al. 2000). These transparent, water‐soluble compounds were first discovered in jellyfish (Wittenberg 1960) and then in red algae (Tsujino 1961) and corals (Shibata 1969). The role of MAAs as UV sunscreens is well established, although other functions, such as antioxidation, have also been discussed for MAAs in the microbial world (Oren and Gunde‐Cimerman 2007, Singh et al. 2008). MAA UV sunscreen activity has been examined in the dinoflagellate, *Gymnodinium sanguineum* (Neale et al. 1998). In dinoflagellates, *Alexandrium* and *Heterocapsa*, it is thought that MAAs may be concentrated around UV‐sensitive organelles (Laurion et al. 2004); however, this has yet to be confirmed.

Fifteen MAAs have been identified in reef‐building coral holobionts (Shick and Dunlap 2002, Rosic and Dove 2011). However, it remains unclear which organism produces these diverse MAAs, since genes and enzymes for their biosynthesis had not been identified in 2009. It has been reported that MAA concentrations in coral holobionts are higher when UV radiation is doubled, but the mechanism of regulation is unknown (Shick 2004). Although it has been hypothesized that MAAs are synthesized via the shikimate pathway (Shick et al. 1999), which includes a plastid‐targeted fusion protein in dinoflagellates (Waller et al. 2006), this remains to be conclusively demonstrated (Cardozo et al. 2007).

The MAA biosynthetic gene cluster was first identified in a cyanobacterium and all four biosynthetic enzymes were characterized in vitro (Balskus and Walsh 2010). Dimethyl 4‐deoxygadusol (DDG) synthase, *O*‐methyl‐transferase (*O*‐MT), and ATP‐grasp are conserved in the cyanobacterial gene cluster. The gene for the fourth biosynthetic step is either a nonribosomal peptide synthetase (NRPS) homolog or a D‐alanine (D‐Ala) D‐Ala ligase homolog. Gene clusters have been also found in red algal genomes and four putative genes for MAA porphyra‐334 are fused into two genes, DDG/*O*‐MT and ATP‐grasp/D‐Ala D‐Ala ligase (Brawley et al. 2017). Five MAAs, mycosporine‐glycine, mycosporine‐2‐glycine, shinorine, palythine, and porphyra‐334, have been identified in the Symbiodiniaceae (Banaszak et al. 2006, Rosic and Dove 2011). At that time, it was predicted that symbiotic algae synthesize the MAAs. However, draft genomes of corals suggest that MAAs are also produced by corals (Shinzato et al. 2011, Bhattacharya et al. 2016). Considering that 15 MAAs have been identified in coral holobionts, our understanding of the enzymatic and genetic basis of their synthesis is still limited. Many homologs for MAA biosynthetic genes have been also reported in transcriptomes of the Symbiodiniaceae (Silva Lima et al. 2020). Each of those genes may be involved in biosynthesis of various MAAs. Omics data and functional analyses will clarify diverse MAA biosynthetic pathways and their regulation (Meyer and Weis 2012).

## THE MAA BIOSYNTHETIC GENE CLUSTER IN BLEACHING‐TOLERANT SPECIES

**Fig. 2:**
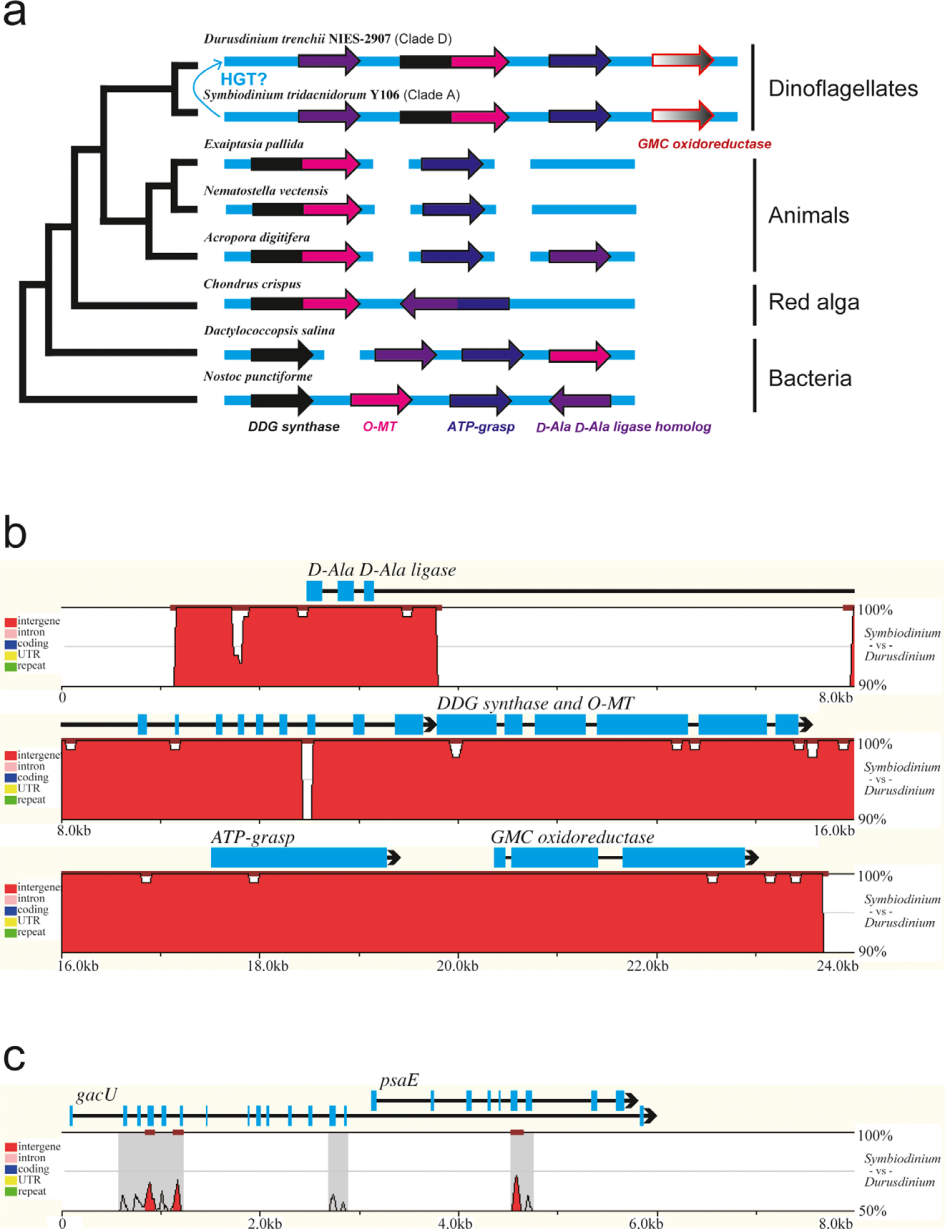
The highly conserved mycosporine‐like amino acid (MAA) biosynthetic gene cluster region that has been found in genomes of *Symbiodinium tridacnidorum* and *Durusdinium trenchii*. (a) A possible phylogenetic relationship for the MAA‐GMC gene cluster is based on the phylogenetic tree of DDG synthase family (Shoguchi et al. 2021). (b) A smooth‐graph from alignments of MAA biosynthetic gene cluster regions between *S. tridacnidorum* and *D. trenchii*. BlastZ alignment and visualization were performed on the zPicture website (Ovcharenko et al. 2004; https://zpicture.dcode.org). The X‐ and Y‐axes are the nucleotide sequence length of *D. trenchii* and the percent identity (ID) between *S. tridacnidorum* and *D. trenchii*, respectively. Default parameters (>100 bp/>70% ID) show conserved elements (red). *D. trenchii* transcriptomes, TRINITY_DN38977_c0_g3_i5, TRINITY_DN52098_c0_g1_i1, TRINITY_DN25387_c0_g1_i1, and TRINITY_DN29905_c0_g1_i1, are mapped as *GMC oxidoreductase*, *ATP‐grasp*, *DDG synthase and O‐MT*, and *D‐Ala D‐Ala ligase*, respectively (Koyanagi et al. 2013, Shoguchi et al. 2021; https://marinegenomics.oist.jp/symbd/viewer/info?project_id=102). Gene structures with exons (blue boxes), introns (black lines) and directions (arrows) are shown on the upper side. Some introns and intergenic regions also show 100% identity, in addition to exons. (c) An alignment of *gacU* and *PsaE* regions is shown for comparison. The *D. trenchii* transcriptomes, TRINITY_DN21134_c0_g1_i1 and TRINITY_DN31838_c0_g2_i1, are mapped for *gacU* and *psaE*. Sequence regions with more than 80% identity are not found, although a few conserved elements are found in exons (>50 bp/>70% ID).

In prokaryotes, conserved MAA biosynthetic gene clusters have been identified (Balskus and Walsh 2010) and the gene cluster in eukaryotes was recently identified in the red alga, *Porphyra umbilicalis* (Brawley et al. 2017). In dinoflagellates, the draft genome of *Symbiodinium tridacnidorum* (previously *Symbiodinium* sp. clade A3) identified a gene cluster for enzymes involved in MAA biosynthesis (Shoguchi et al. 2018). The common characteristic of red algae and dinoflagellates was a possible fused protein with functions of both DDG synthase and *O*‐MT (Waller et al. 2006). The fused protein is also predicted in genomes of sea anemones and corals, but evolutionary relationships and functions remain to be examined. Comparative analysis suggests that orthologs of these genes have been lost in the common ancestor of *Breviolum* and *Cladocopium*, although homologs for MAA biosynthetic genes have been described in genomic analyses of *C. goreaui* (Liu et al. 2018). Other genes for UV‐damage protection may exist in the *Breviolum* and *Cladocopium* genomes (Liu et al. 2018, Shoguchi et al. 2018). However, an alternative strategy among crown groups of Symbiodiniaceae likely utilizes sunscreens from the hosts, because some hosts, including sea anemones and corals, also have gene homologs for MAA biosynthetic genes (Fig. 2a). In addition, cnidarians have diverse fluorescent proteins with photoprotective functions (Matz et al. 1999, Alieva et al. 2008, Smith et al. 2013, Satoh et al. 2020, Kashimoto et al. 2021). Thus, it is possible to speculate that late‐diverging groups, *Breviolum* and *Cladocopium*, require hosts such as cnidarians to receive adequate insolation. Genome evolution of the Symbiodiniaceae has been discussed (González‐Pech et al. 2019). The loss of gene families for metabolic pathway genes may contribute to the transition from free‐living to putative obligate symbionts (Shoguchi et al. 2018, González‐Pech et al. 2019).

On the other hand, the draft assembly of *Durusdinium trenchii*, which occupies an intermediate phylogenetic position, contains a gene cluster for MAA biosynthesis, expression of which is supported by transcriptomic data (Shoguchi et al. 2021; Fig. 2b). Moreover, the gene order is conserved between *Symbiodinium tridacnidorum* and *D. trenchii*. Additionally, we found that the neighboring gene to *ATP‐grasp* on the 3′‐end of the cluster encodes an enzyme resembling a member of the GMC (glucose‐methanol‐choline) oxidoreductase family. This homolog also occurs in the genome of *S. tridacnidorum* and is located adjacent to the MAA gene cluster (Fig. 2), indicating syntenic conservation of metabolic genes among members of the Symbiodiniaceae (Liu et al. 2018). It has been suggested that GMC oxidoreductase with FAD may change enzyme activity by altering light intensity (Shoguchi et al. 2021), but it remains unknown whether this GMC oxidoreductase functions in MAA biosynthesis. As far as I am aware, the MAA‐GMC gene cluster has not been studied in cyanobacteria and other organisms (Singh et al. 2010). Functions of proteins and regulatory mechanisms in MAA biosynthesis with potentially polycistronic and alternative mRNAs will be clarified by hetelorogous expression analysis.

## POSSIBLE RECENT HORIZONTAL TRANSFERS OF MAA BIOSYNTHETIC GENES AMONG TAXA OF THE SYMBIODINIACEAE

Predicted fusions of *DDG synthase* and *O‐MT* suggest that dinoflagellates obtained the fused gene from a red alga through secondary endosymbiosis (Waller et al. 2006, Brawley et al. 2017). The *DDG synthase* of *Symbiodinium tridacnidorum* Y106 may also have been transferred from a red alga, according to molecular phylogenetic analysis (Shoguchi et al. 2018; Fig. 2a). On the other hand, molecular phylogenetics for the GMC oxidoreductase of the Symbiodiniaceae found that evolutionary relationships remain uncertain (Shoguchi et al. 2021). Therefore, I cannot exclude the possibility that the *MAA‐GMC* cluster formed in the Symbiodiniaceae lineage. For example, sequences similar to reverse transcriptases, related to retroviruses or retrotransposons, were found ∼9 kbp downstream of the *MAA‐GMC* cluster in *S. tridacnidorum*, suggesting the involvement of transposition mechanisms. Since Symbiodiniaceae genomes have repeated transposon sequences (Song et al. 2017), understanding formation of this cluster is likely to be an interesting research topic in dinoflagellate genome evolution (Hou et al. 2019).

*Durusdinium trenchii* NIES‐2907 genome sequences imply putative conservation of the *MAA‐GMC* cluster (genes for D‐Ala D‐Ala ligase, DDG synthase, *O*‐MT, ATP‐grasp, and GMC oxidoreductase) between *Symbiodinium tridacnidorum* and *D. trenchii* (Shoguchi et al. 2021). Available transcriptomic data from other Symbiodiniaceae strains (Keeling et al. 2014, Yu et al. 2020) suggest that *S. tridacnidorum* CCMP2430 (transcriptome assemblies: MMETSP1115, MMETSP1116, and MMETSP1117) and *D. trenchii* (transcriptome assembly: MMETSP1377) have highly conserved genes for D‐Ala D‐Ala ligase, DDG synthase, *O*‐MT, ATP‐grasp, and GMC oxidoreductase (data not shown). The possibility of cross‐contamination in laboratory cultures may be discussed when genomes of those strains with transcriptomic information are decoded in the future. Curiously, pairwise alignments of *MAA‐GMC* clusters include intron and intergenic sequences with 100% matches between *S. tridacnidorum* (scaffold 314) and *D. trenchii* (scaffold 2498). *MAA‐GMC* cluster regions, including introns and intergenic sequences, are highly conserved between *Symbiodinium* and *Durusdinium*. Sequence similarities of ∼20 kb were >99%, except for the third intron of *D‐Ala D‐Ala ligase*. The third intron of *D‐Ala D‐Ala ligase* had an insert of ∼4 kb in *D. trenchii*. The only *D‐Ala D‐Ala ligase* has typical exon‐intron structure, in contrast to other genes with long exons (Fig. 2b). On the other hand, an alignment of *S. tridacnidorum* scaffold 4185 and *D. trenchii* scaffold 331, which contains a microsyntenic region, including genes from secondary endosymbiosis, showed less than 80% similarity of *gacU* (*Rho GTPase‐activating protein*) and *psaE* (*Photosystem I reaction center subunit IV*; Hackett et al. 2004, Mungpakdee et al. 2014). Apparent homology of exons was confirmed (Fig. 2c), corresponding to comparative analyses of transcriptomes with 72.7–81.5% similarity (Ladner et al. 2012). Therefore, it is difficult to explain sequence conservation of MAA biosynthetic gene regions during the ~160 my since their divergence. I hypothesize that HGTs of the gene cluster may have occurred among Symbiodiniaceae species, although more evidence for it is required (Fig. 1a). If there were no HGT events among Symbiodiniaceae species, how have conserved sequences, which include noncoding sequences (introns and intergenic sequences) been maintained in both lineages? To find the conserved *MAA‐GMC* cluster, the genome of the other *S. tridacnidorum* CCMP2592 from the Great Barrier Reef (GBR) and the other *Symbiodinium* genomes (González‐Pech et al. 2021, Yoshioka et al. 2021) were surveyed, but the cluster was not detected (data not shown). This supports the genomic structural divergences that have been reported in the genus *Symbiodinium* (González‐Pech et al. 2021). In the case of *S. tridacnidorum* and *D. trenchii* (Shoguchi et al. 2018, 2021), both strains are from Okinawa, Japan, suggesting that detailed syntenic analysis of Symbiodiniaceae genomes from the same habitats may detect additional highly conserved noncoding sequences (>200 bp), which could be defined as ultraconserved elements (100% identity with no insertions or deletions; Bejerano et al. 2004). On the other hand, this hypothesis does not exclude the possibility of convergent evolution, because the highly conserved intragenic and intergenic regions may be important for regulation or efficient transcription of clustered genes. High‐quality genomes likely will detect sequences for increasing numbers of HGT events (Van Etten and Bhattacharya 2020, Marinov et al. 2021, Nand et al. 2021) and ultraconserved elements. Both *S. tridacnidorum* Y106 and *D. trenchii* NIES‐2907 have been found in the same habitat (Shoguchi et al. 2018, 2021), implying possible HGT. Thus, Symbiodiniaceae–Symbiodiniaceae interactions will be also investigated in holobiont genome analyses (Matthews et al. 2020, McIlroy et al. 2020), in addition to Symbiodiniaceae–bacteria connections (Garrido et al. 2020). Furthermore, it remains to be seen whether HT genes in dinoflagellates depend on mechanisms similar to those of bacteria (Thomas and Nielsen 2005, McDaniel et al. 2010, Lang et al. 2012, Soucy et al. 2015) or associated viruses (Correa et al. 2013). For examples of possible HT genes, a Form II RuBisCO from proteobacteria and DVNPs (dinoflagellate/viral nucleoproteins) from an algal virus have been reported in dinoflagellates (Morse et al. 1995, Gornik et al. 2012, Janouškovec et al. 2017). HGTs may be more common in cases of reduced adaptability when the capacity for sexual reproduction is reduced (Brian et al. 2019, Shah et al. 2020).

## PERSPECTIVES

Draft genomes from the Symbiodiniaceae have revealed many unusual gene structures in their nuclear genome sequences that are complicated by an abundance of duplicate genes, spliceosomal introns, and transposable elements (Lynch and Conery 2003). When findings of MAA biosynthetic clusters in Symbiodiniaceae genomes were reviewed, one question was whether acquisition of the MAA biosynthetic gene cluster by the Symbiodiniaceae lineage led to higher bleaching resistance. In addition, it remains unexamined whether clustered gene structures in dinoflagellate genomes enhance their transcriptional efficiency (Stephens et al. 2020). The MAA‐GMC gene cluster has been found only in two genomes in the Symbiodiniaceae, probably due to sampling bias in genome sequencing. Since sunlight intensity in seawater is important for survival in coral reefs, multiple tools to cope with it have likely evolved in symbiotic dinoflagellates (Maruyama et al. 2015, Shimakawa et al. 2021). Little is known about how symbioses between corals and Symbiodiniaceae populations respond to dramatic climate shifts, but trade‐offs between fast growth and thermal tolerance have been discussed (van Oppen and Medina 2020). If both hosts and dinoflagellates can produce MAAs, coral holobionts, including potentially opportunistic or parasitic *Durusdinium* may have become more adaptable (LaJeunesse et al. 2009, Lesser et al. 2013).

I propose that a gene cluster for MAA biosynthesis in Symbiodiniaceae genomes may function as a tool for bleaching resistance, if diversification of the capacity for MAA biosynthesis partially contributes to maintenance of symbiotic relationships in response to UV stress (Fig. 1b). To test this hypothesis, the mechanism of HGTs will be explored by functional analyses of sequences adjoining the conserved *MAA‐GMC* cluster. If HGTs between Symbiodiniaceae genomes can be induced in the lab, possible gene sets for bleaching resistance may be validated in the near future. It has been reported that *Symbiodinium* (ITS type: A3) and *Durusdinium* (ITS type: D1a) increased during rapid recovery from bleaching in the Caribbean (Kemp et al. 2014). During recovery, possible gene sequences for bleaching resistance may be detected in environmental DNA. Other thermally tolerant species have been reported in addition to possible bleaching‐tolerant symbionts (Swain et al. 2017, Ziegler et al. 2017, LaJeunesse et al. 2018). Those include GBR populations of the generalist *Cladocopium* (ITS type: C1; Howells et al. 2011, Levin et al. 2016) and *Cladocopium thermopilum* (C3gulf) in the Persian Gulf (Hume et al. 2016, Howells et al. 2020). Have any of those populations acquired functional genes via HGTs or epigenetic changes that are involved in thermal tolerance? It remains to be seen whether the ability to synthesize MAAs has increased in thermally tolerant species. Biosynthesis of other metabolites and gene functions that I did not review may prove more important than MAA biosynthesis for bleaching resistance (Bellantuono et al. 2019, Poquita‐Du et al. 2020, Yuyama et al. 2021). Omics resources with major‐minor symbiont interactions will likely provide further insights into the relationship between symbionts and hosts (Weber and Medina 2012, Shinzato et al. 2014a, Weis 2019, González‐Pech et al. 2021, Williams et al. 2021).

## Author Contribution

**E. Shoguchi:** Conceptualization (lead); Funding acquisition (lead); Resources (lead); Supervision (lead); Visualization (lead); Writing‐original draft (lead); Writing‐review & editing (lead).
